# The Hospitals and the Wounded: How to Safeguard Civilian Patients

**Published:** 1915-01-09

**Authors:** 


### THE HOSPITALS AND THE WOUNDED.

### How to Safeguard Civilian Patients.

To all concerned it has been a source of satisfaction that our civil hospitals have been ready and willing to take an active part in the care and treatment of our wounded soldiers. From the very commencement of the war most of them, if not all, expressed to the authorities their desire to place a certain proportion of their beds at the disposal of the War Office or Admiralty, and they have more than redeemed their promise. We suppose there is not either in London or elsewhere a hospital of any importance which has not gladly welcomed and treated a greater or less number of sick and wounded men. No one would have it otherwise, for the men deserve everything which is due to those who have taken the supreme risk for our homes, our liberties, and our lives. Whatever is necessary for their welfare and recovery they must have in spite of price and consequence.

### The Civil Population.

When the arrangements now in operation were first contemplated we pointed out in these columns that the reception of wounded men into civil hospitals must inevitably mean a restriction of the hospital facilities of the ordinary population; and we urged the managers of the hospitals to endeavour to make arrangements to meet this position. Some of them have been able to do so, but in other instances the question of expense has made such a step impossible. In these latter instances, therefore—and we fear they are the majority—the arrival of the wounded has excluded from hospital treatment a certain number of civil patients who in ordinary times would have been admitted. This is unfortunate and is to be regretted, as everyone will allow. But in the circumstances it is inevitable, and we must accept it as inevitable. We would rather that no one was excluded, but if there is to be exclusion public opinion would never admit that it is the soldiers who must suffer. Their claim in a great national emergency such as the present is of the first rank and prior to all others. This attitude of mind, however natural, begs the question from the voluntary hospital point of view, since it is not primarily their business to attend other than civilian men and women, who during sickness are unable to provide satisfactorily for themselves. Thus, in typical English fashion a compromise has been effected; but like all compromises it is a delicate matter, and we must not do all we can for the soldier while leaving the civilian unprovided for. But if it is admitted, as it must be, that the existing arrangements—necessary though they be—involve hardship, it is obviously desirable that this hardship shall be as limited as is possible. We want, in other words, while doing full justice to the soldiers, to avoid overlooking the wants of other sufferers. If at this moment we recall these latter it is not that we are not sensible of the difficulties both of the hospital governors and of the military authorities.

It is quite impossible to say what accommodation is necessary for wounded soldiers at any given moment. Almost on any day these may arrive in considerable numbers, and for all of them beds must somehow or other be provided. To prescribe some hard-and-fast rule is clearly impossible, and in these circumstances it must sometimes happen that in one or more of the civil hospitals beds are for a time left unoccupied. All this we fully recognise, but at the same time we wish to ask whether something may not be done to mitigate the prejudice which civilian patients are undoubtedly suffering. If we must admit, as we fear is necessary, that when financial claims are so numerous it is hopeless to enlarge the existing hospital accommodation, there is the more necessity for making the best and most complete use of what we possess.

### A Word to Hospital Managers.

We therefore venture to urge on hospital managers—and we may say on the staffs also—the obligation of a very active supervision in reference to the admission and retention of patients. The circumstances make these considerations just now of paramount importance. They are of importance at all times, but they have a truly acute significance in the present emergency. Secondly, we suggest that where beds, though earmarked for soldiers, are kept empty for a time, the hospital managers ought to communicate with the military authorities with a view to see whether a less provision will suffice. The difficulties of these authorities are great, but so also are the sufferings of members of the poorer classes who in consequence of the present position are denied the benefits of hospital treatment. To consider these is not the duty of the War Office, but it is the duty of the hospitals. This duty must be performed, even though its discharge may seem to add to the embarrassment of officers whose shoulders already carry heavy burdens.

### The Need for Co-ordination.

The fact of the matter is that it must be regarded as a civic obligation to urge that the civil hospitals shall not be invaded more than is absolutely necessary to do justice to those who have suffered in their country's cause, and that only in so far as the military hospitals fail shall accommodation be taken from the general community. We think these ends would be more easily attained were some co-ordinated action secured between the Army representatives and the hospital managers. It is not easy to make new arrangements in times of stress, but we feel sure some such plan as is here suggested must be adopted if unnecessary suffering is to be avoided among the poorer classes of our people.

